# Stimulant use in medical students and residents requires more careful attention

**DOI:** 10.22088/cjim.9.1.87

**Published:** 2018

**Authors:** Golnaz Fallah, Sussan Moudi, Angela Hamidia, Ali Bijani

**Affiliations:** 1 1.Student Research Committee, Babol University of Medical Sciences, Babol, Iran; 2 2.Cancer Research Center, Health Research Institute, Babol University of Medical Sciences, Babol, Iran; 3 Department of Psychiary, Babol University of Medical Sciences, Babol, Iran; 4 Social Determinants of Health Research Center, Health Research Institute, Babol University of Medical Sciences, Babol, Iran; 5 Non-Communicable Pediatric Diseases Research Center, Health Research Institute, Babol University of Medical Sciences, Babol, Iran

**Keywords:** Stimulants, Medical students, Residents

## Abstract

**Background:**

Stimulant pharmaceuticals are abused among academic students to elevate mood, improve studying, intellectual capacity, memory and concentration, and increase wakefulness. This study was designed to evaluate the current situation of stimulant use among medical students and residents of Babol University of Medical Sciences.

**Methods:**

This cross-sectional study was conducted among 560 medical students and clinical residents of Babol University of Medical Sciences during the academic year 2014-2015. A self-administered questionnaire was used for data collection.

**Results:**

Four hundred and forty-four (79.3%) students filled out the questionnaires. 49 (11%) individuals reported amphetamine and methylphenidate (ritalin) use. The mean age of the stimulant drug users was 24.6±4.8 years. The main initiator factor was to improve concentration (29 persons; 59.2%). There were significant statistical correlations between stimulant drugs abuse and male gender, living in dormitor in residence and internship and past medical history of psychiatric disorders (depression, attention deficit hyperactivity disorder and substance abuse) (p<0.05).16 (32%) students started the drug use on their friends’ advice; 15 (30%) due to self-medication and 12 (24%) persons with physician´s prescription.

**Conclusions:**

Because of significant prevalence of stimulant use, regulatory governmental policies and also planning to improve essential life skills, awareness about the side effects and complications of these drugs, screening of at-risk college students and early identification of the abusers are suggested.

Medical students are potentially at higher risk of non-medical prescription use of stimulant drugs (methylphenidate and amphetamines) related to their academic conditions such as prolonged wakefulness, series of professional examinations as indicators of their competence, stress, need to enhance their ability to focus and concentrate in writing and studying; and finally, improve their academic performance (1-3). Drugs like methylphenidate (ritalin) and amphetamines are common pharmaceuticals in access which their sympathomimetic effects such as elevating mood, enhancing wakefulness, improveing memory, concentrating and learning, as the leading factors to increase the prevalence of use within college students and adults (2,4). Stimulants, such as methylphenidate, act in the brain similarly to brain neurotransmitters include norepinephrine and dopamine. Stimulants enhance the effects of these chemicals in the brain. The associated increase in dopamine can induce a feeling of euphoria; they also increase blood pressure and heart rate, constrict blood vessels, increase blood glucose, and open up breathing passages (5-8). Multiple studies examined the extent and different aspects of these stimulant use in general healthy populations and college students during the past few years (1-4, 9-19).

The systematic review literature of Finger (2) revealed that prevalence of Ritalin use among medical students reached 16%, and the most common route (95.3%) of administration was ingestion. In a case-series study performed in Iran among a small sample size of medical students, it was mentioned that average illicit drug abusers was high in participants (11). The aim of this study is to determine the prevalence of stimulant use in medical students and residents; the results can help the policy- makers for planning and implementing interventional approaches. 

## Methods

This analytic observational analytical cross-sectional study was performed among medical students and residents of Babol University of Medical Sciences, Babol, North of Iran in the academic year 2014-2015. All clinical residents and medical students (from the 1^st^ to the last academic year), studying or working in the hospitals affiliated to Babol University of Medical Sciences, were invited to participate in the study. Data collection was performed with a self-administered questionnaire in which its validity and reliability were confirmed through an expert panel. Demographic characteristics and the condition of stimulant use (included start time, the route, duration and causes of administration) were assessed. All of the students were informed about the objectives of the study, and participated voluntarily, as well as confidentiality was assured to them. This study was approved by the Research Committee of Babol University of Medical Sciences. The collected data were analyzed by SPSS package Version 16. Chi-square test was used to assess the correlation between quantitative variables and independent t-test for comparison between the two groups with and without use of stimulant drugs. Results were analyzed at the significant level of 95%. 

## Results

Five hundred and sixty questionnaires were distributed among the medical students and residents. Four hundred and forty-four participants filled out the questionnaires (participation rate: 79.3%); 260 (58.6%) females and 184 (41.4%) males. 98 (22.1%) in the first 5 semesters of academic year (basic course), 66 (14.9%) in physiopathology, 101 (22.7%) clerkship, 120 (27.0%) internship and 59 (13.3%) individuals in residency course. The mean age of participants was 23.7±4.8 years; most of them (79.5%) were unmarried and 41.2% were living with their family. Their demographic characteristics of studied subjects are presented in table 1. 

**Table 1:** Distribution of demographic variables

**Variable**	**Number (%)** **n=444**
Marital status	
Single Married Divorced or widowed	353 (79.5) 89 (20) 2 (0.5)
Place of living	
Dormitory Living with family Individual home	169 (38.1) 183 (41.2) 92 (20.7)
Familial history of psychiatric disorders	
Yes No	23 (5.2) 421 (94.8)
Past history of referring to a psychiatrist psychologist	
Yes No	33 (7.4) 411 (92.6)

Most of the students did not have the past medical history or familial history of psychiatric disorders or past history of referring to a psychiatrist or a psychologist. Forty-nine (11%) individuals reported stimulant use: 29 (6.5%) participants used ritalin while 11 (2.5%) consumed amphetamine. Among the students who recorded the cause, start time, the route and dose of drug administration, 29 (59.2% of abusers) individuals used these drugs for enhancing focus and concentration, 3 (6.1%) for increasing self-esteem and 3 (6.1%) for elevating mood. 16 (32.7% of users) individuals have started the use during recent one year period 11 (22.4%) in the last 6 months, and 3 (6.1%) for the past one month. The route of administration was ingestion in 39 (79.6% of users) students, 3 (6.1%) intravenous and 1 (2%) was inhalation. Out of 29 ritalin users 15 (51.7%) subjects, started on a daily dose of 5mg, 10 mg/day in 3 (10.3%) subjects 2.5 mg/day in 2 (6.9%) individuals; and 9 subjects (31%) recorded a daily dose of more than 10 mg. Most of them (32%) started the use of drugs due to their friends´ advice. Other reported reasons for stimulant initiation were self-medication (30%) and physician´s prescription (24%). The correlation of stimulant use with demographic and psychiatric characteristics of medical students is presented in table 2. There were significant associations between stimulant use and gender, the place of living and educational level (p<0.05). The prevalence rates of ritalin use were 3.1%, 6%, 3.9%, 8.3% and 13.5% comprising basic course, physiopathology, clerkship, internship and residency course, respectively (p<0.001). 

**Table 2:** The association of stimulant use with demographic and psychiatric characteristics of medical students

**Variable**	**With stimulant use** **n=49** **N (%)**	**Without stimulant use** **n=395** **N (%)**	**p-value**
Age (mean±SD)	24.6±4.8	23.6±3.9	0.12
Gender			
Male Female	29 (59.2) 20 (40.8)	155 (39.2) 240 (60.8)	0.009
Marital status			
Without spouse With spouse	40 (81.6) 9 (18.4)	315 (79.7) 80 (20.3)	0.852
The place of living			
Dormitory Living with family Individualized home	27 (54.7) 11 (23.4) 9 (19.1)	41 (35.7) 171 (43.3) 83 (21)	0.009
Academic level			
Basic course Physiopathology Clerkship Internship Residency	5 (10.2) 18 (36.7) 4 (8.2) 14 (28.6) 8 (16.3)	93 (23.5) 48 (12.2) 97 (24.6) 106 (26.8) 51 (12.9)	0.001

## Discussion

In this research, 11% of medical students and clinical residents had ritalin or amphetamine use. This result is somewhat different with the past similar studies; in the study of Finger (1) by analyzing all articles published between 1990 and 2012 in English, Portuguese, and Spanish, the prevalence of methylphenidate use in medical students represented 16%; while in the research of Laporte in Spain (16), 22% out of 102 medical students had more than one time amphetamine use in the past 6 months; and finally in the research of Khademi in Tehran University of Medical Sciences, Iran (14) among 240 clinical residents, 23% had ritalin use in the past one year and 6.6% in the past one month. These differences with regard to the prevalence of stimulant use can be correlated to different geographical locations and other characteristics of the study population and different levels of access to these substances. Besides, in this research, we used a self-administered questionnaire for data collection and did not check them with laboratory diagnostic tests. On the other hand, amphetamine use is illigal in Iran; therefore, the students might refuse to express the real condition. 

In our study, stimulant use was more prevalent in males; a significant difference was observed between two sexes (p=0.009); that is in contrast to Finger´s study results (2) in which no gender difference was found, also consistent to the research of Khademi (14) where males are relatively likely to use ritalin.

The common ways that medical students started stimulant use were self- medication or friends’ influence. Also, in Valipour’s research in Iran (17), 50% of college students mentioned that their tramadol or ritalin use to seek pleasure was due to peer suggestion. The main contributing factors for starting drug abuse are peer pressure (20, 21), and the easy access to these substances (20). Therefore parents should be aware of their kids’ bchavior and the relationship they have with their friends to prevent unwanted substance abuse. In this research, there is a significant difference in the prevalence of stimulant use between the medical students who live in dormitories compared to those who live with their family or individual home; it means that providing entertainment for students living in dormitories is such as putting of audiovisual and electronic facilities, sports and so to spend their time productively. In this study, most of stimulant consumers started these drugs in the recent one-year that is consistent with Finger´s study (2) which mentioned that most of medical students began using the drug after entering the university. There was a significant association between educational level and stimulant use; stimulant use was more common in higher academic level (p<0.001). Increasing the concentration and prolonged wakefulness were presented as the main causes of stimulant use. These results can be due to multiple-related factors. The academic exam for clinical residency is rigorous and competitive; likewise stressful higher-level comprehensive exams in General Medicine need more focus and concentration, satisfactory academic practice and studying; and these factors can lead the candidates to use stimulant drugs. It shows that decision-makers should plan and implement continuous training programs to improve essential life-skills such as decision making refusal skills and problem solving skills. Finger´s study (2) showed no evidence that the use of methylphenidate was beneficial in terms of memory or learning and it was concluded that ritalin simply increased wakefulness and alertness, reducing the time of sleep. In a recent study in Canada (10), it has been mentioned that neither drug efficacy, nor benefit-to-risk balance, nor indicators of current or growing demand provided sufficient evidence that methylphenidate could be considered as a suitable example of a cognitive enhancer. Thereby these results should be presented to all of medical students, especially in higher academic levels. 

The most important limitations of this research are data collection using a self-administered questionnaire, not performing a structured clinical interview for diagnosis of comorbid psychiatric disorders and not examining the current use of stimulant drugs with diagnostic laboratory tests. 

In conclusion,** c**onsidering the significant prevalence of stimulant use in medical students, regulatory governmental policies and also training programs to promote life- skills and educate theim about the adverse effects of these drugs should be implemented to remedy the extension of stimulant use among medical students.
